# Supplementary material for: Enhancing the Photo and Thermal Stability of Nicotine through Crystal Engineering with Gentisic Acid
Source: Molecules. 2022 Oct 13;27(20):6853. doi: 10.3390/molecules27206853 (PMC9611154; doi:10.3390/molecules27206853)
Supplement: Supplementary file 1 [file molecules-27-06853-s001.zip › molecules-1951883-supplementary.pdf]

Supporting Information: Enhancing the Photo and Thermal Stability of Nicotine Through Crystal Engineering with Gentisic Acid

Devin J. Angevine, Kristine Joy Camacho, Javid Rzayev, and Jason B. Benedict*

Department of Chemistry, University at Buffalo, Natural Sciences Complex, Buffalo, 14260-3000, USA

Supporting Information

Table of Contents

Crystallographic Information 1

Structural Data Deposition 1

(S)-Nicotinium Orotate Hemihydrate Crystals & Crystal Structure 1

Hydrogen Bonding Interaction Table 4

UV Degradation Studies Spectra 5

Hirshfeld Interaction Percentages 10

Thermal Properties 11

IR Spectra 12

PXRD Before and After UV Irradiation 17

Crystallographic Information

Structural Data Deposition

Deposition number 2168648 contains the supplementary crystallographic data for this paper. This data is provided free of charge by the joint Cambridge Crystallographic Data Centre and Fachinformationszentrum Karlsruhe Access Structures service www.ccdc.cam.ac.uk/structures.

(S)-Nicotinium Orotate Hemihydrate Crystals & Crystal Structure

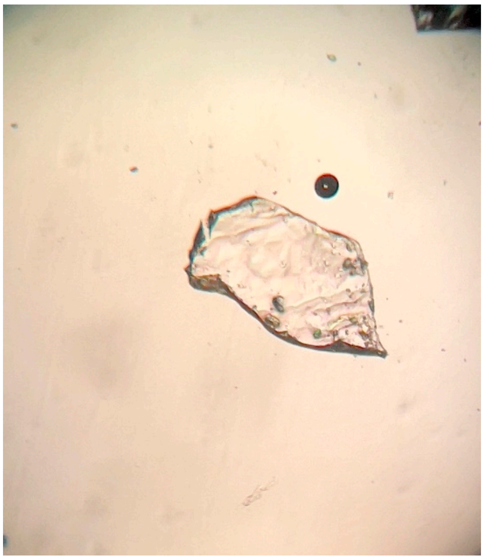

Figure S1: A single crystal of monoclinic $P2_1$ (S)-nicotinium gentisate.

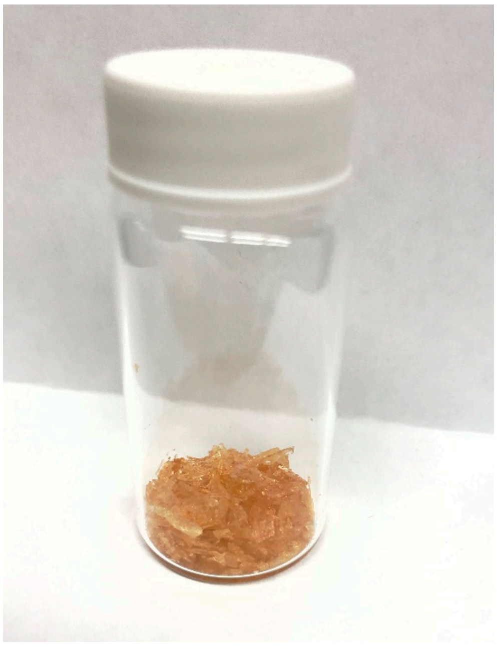

Figure S2: A vial of crystals of monoclinic $P2_1$ (S)-nicotinium gentisate.

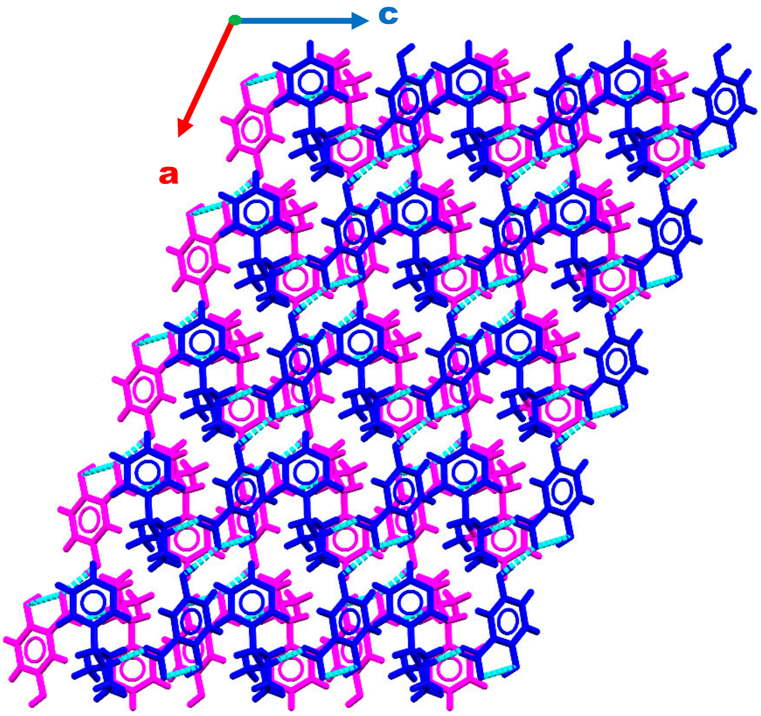

Figure S3: Diagram of monoclinic $P2_1$ (S)-nicotinium gentisate viewed down [010], depicting the H-bonds that are present between wires and sheets of wires .Hydrogen bond interactions highlighted with blue dashed lines.

Table S1: Crystallographic information for nicotinium gentisate.

**Identification code**	**Nicotinium_Gentisate**
**Empirical formula**	$C_{17}H_{20}N_2O_4$
**Formula weight**	316.35
**Temperature/K**	90
**Crystal system**	monoclinic
**Space group**	$P2_1$
**a/Å**	8.2035(3)
**b/Å**	10.9105(4)
**c/Å**	9.2122(3)
**α/°**	90
**β/°**	112.6420(10)
**γ/°**	90
**Volume/Å^3**	760.98(5)
**Z**	2
**ρ_{calc}g/cm^3**	1.381
**μ/mm^{-1}**	0.099
**F(000)**	336
**Crystal size/mm^3**	0.36 × 0.18 × 0.05
**Radiation**	MoKα (λ = 0.71073)
**2Θ range for data collection/°**	4.79 to 63.098
**Index ranges**	-12 ≤ h ≤ 12, -16 ≤ k ≤ 16, -13 ≤ l ≤ 13
**Reflections collected**	15101
**Independent reflections**	5073 [R_{int} = 0.0234, R_{sigma} = 0.0240]
**Data/restraints/parameters**	5073/1/288
**Goodness-of-fit on F^2**	1.056
**Final R indexes [I>=2σ (I)]**	R_1 = 0.0289, wR_2 = 0.0768
**Final R indexes [all data]**	R_1 = 0.0298, wR_2 = 0.0776
**Largest diff. peak/hole / e Å^{-3}**	0.31/-0.21

Hydrogen Bonding Interaction Table

Table S2: Hydrogen bonding interactions with appropriate interaction distances and interactions angles between donor atoms (***D***) and acceptor atoms (***A***). ESDs are shown in parentheses.

**Participating Atoms (*D*—H···*A*)**	***D*—H (Å)**	**H···*A* (Å)**	***D*···*A* (Å)**	***D*—H···*A* (°)**
O4—H4···O1^{i}	0.86(2)	1.82(2)	2.6674(12)	170(2)
O3—H3···O1	0.90(2)	1.71(2)	2.5484(14)	154(2)
C12—H12B···O4^{ii}	0.98	2.43	3.2418(16)	140.0
C10—H10A···O2^{iii}	0.99	2.54	3.4626(16)	154.1
C8—H8A···O4^{ii}	0.99	2.54	3.1310(17)	117.8
C8—H8B···N2^{ii}	0.99	2.47	3.4321(17)	163.4
N1—H1···O2	0.89(2)	1.79(2)	2.6773(13)	176(2)
Symmetry codes: (i) *x*−1, *y*, *z*; (ii) −*x*+1, *y*+1/2, −*z*+2; (iii) −*x*, *y*+1/2, −*z*+1; (iv) −*x*+1, *y*+1/2, −*z*+1.				

UV Degradation Studies Spectra

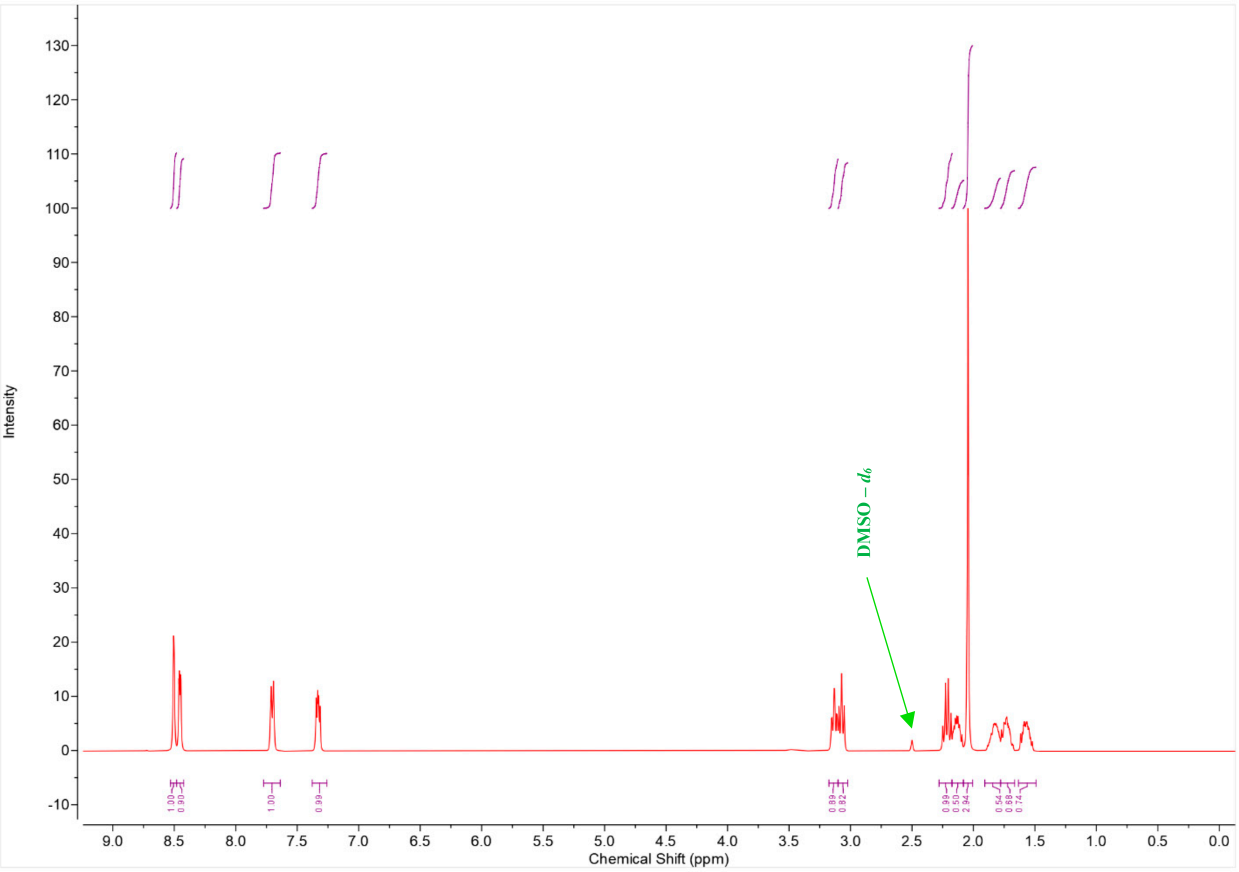

Figure S4: ^{1}H-NMR spectrum for (S)-nicotine from the bottle.

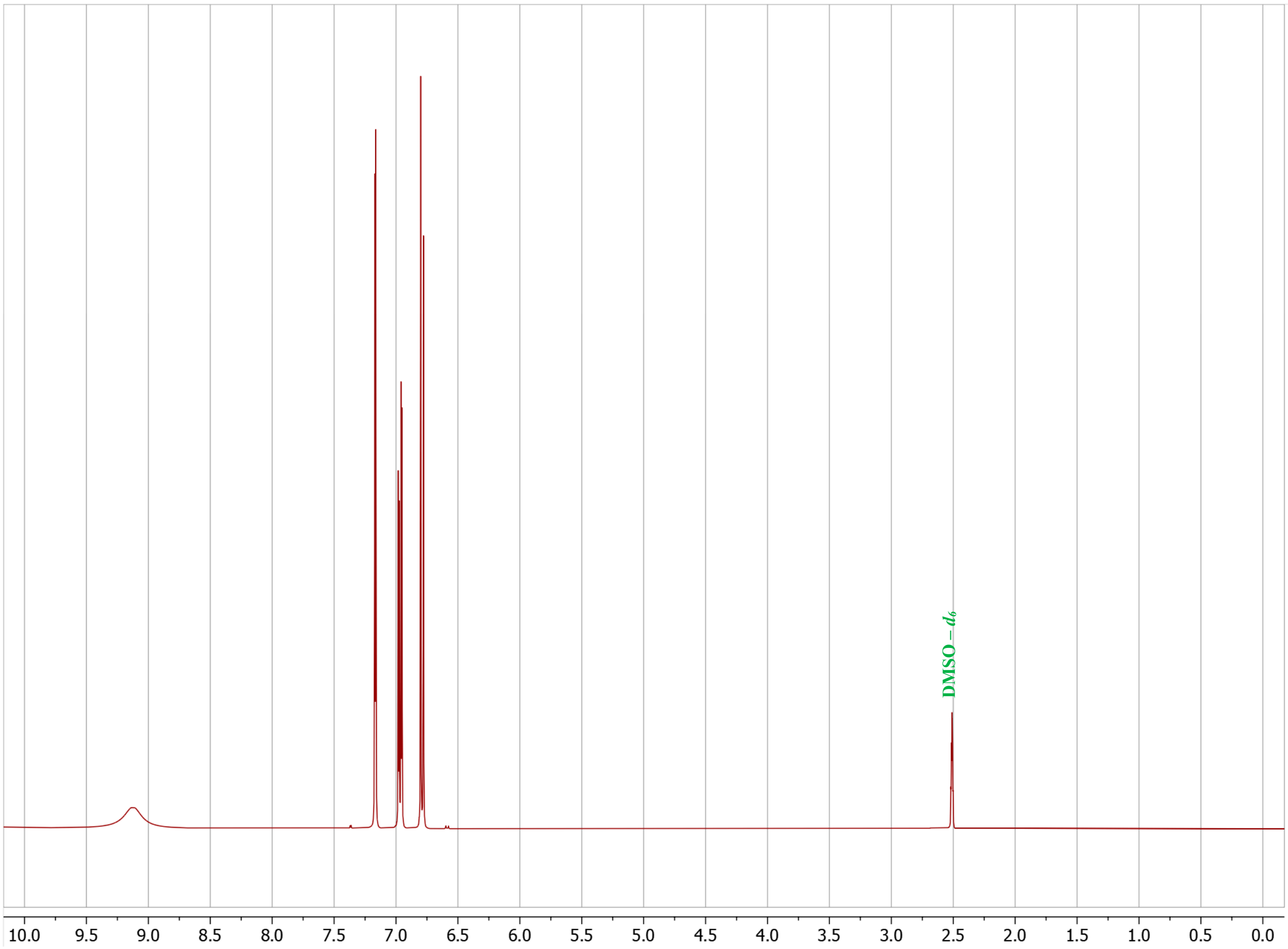

Figure S5: ^{1}H-NMR spectrum for gentisic acid from the bottle.

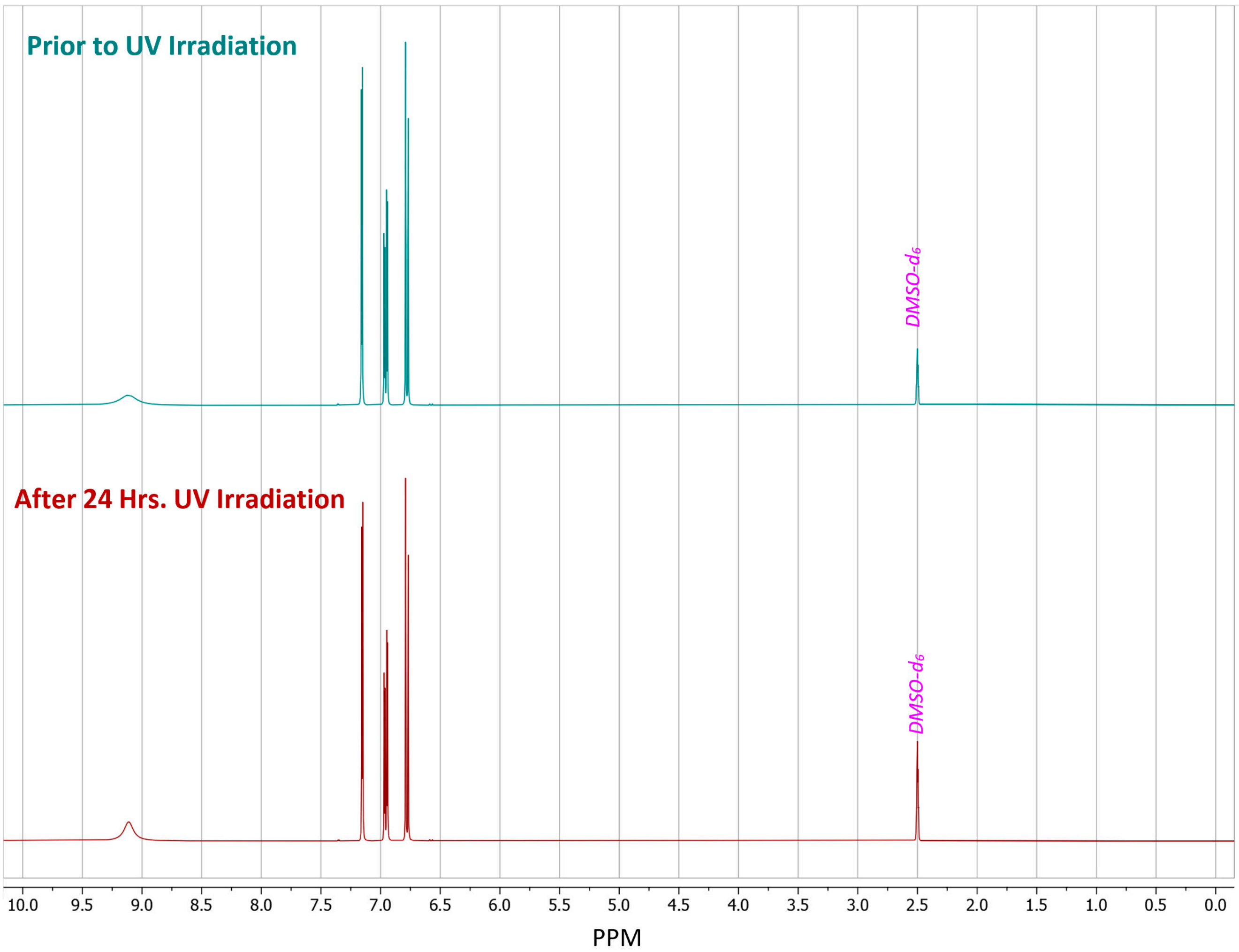

Figure S6: ^{1}H-NMR spectra of solutions of gentisic acid prepared from samples in which the crystalline phase received either no UV irradiation (upper) or 24 hours of UV irradiation (lower).

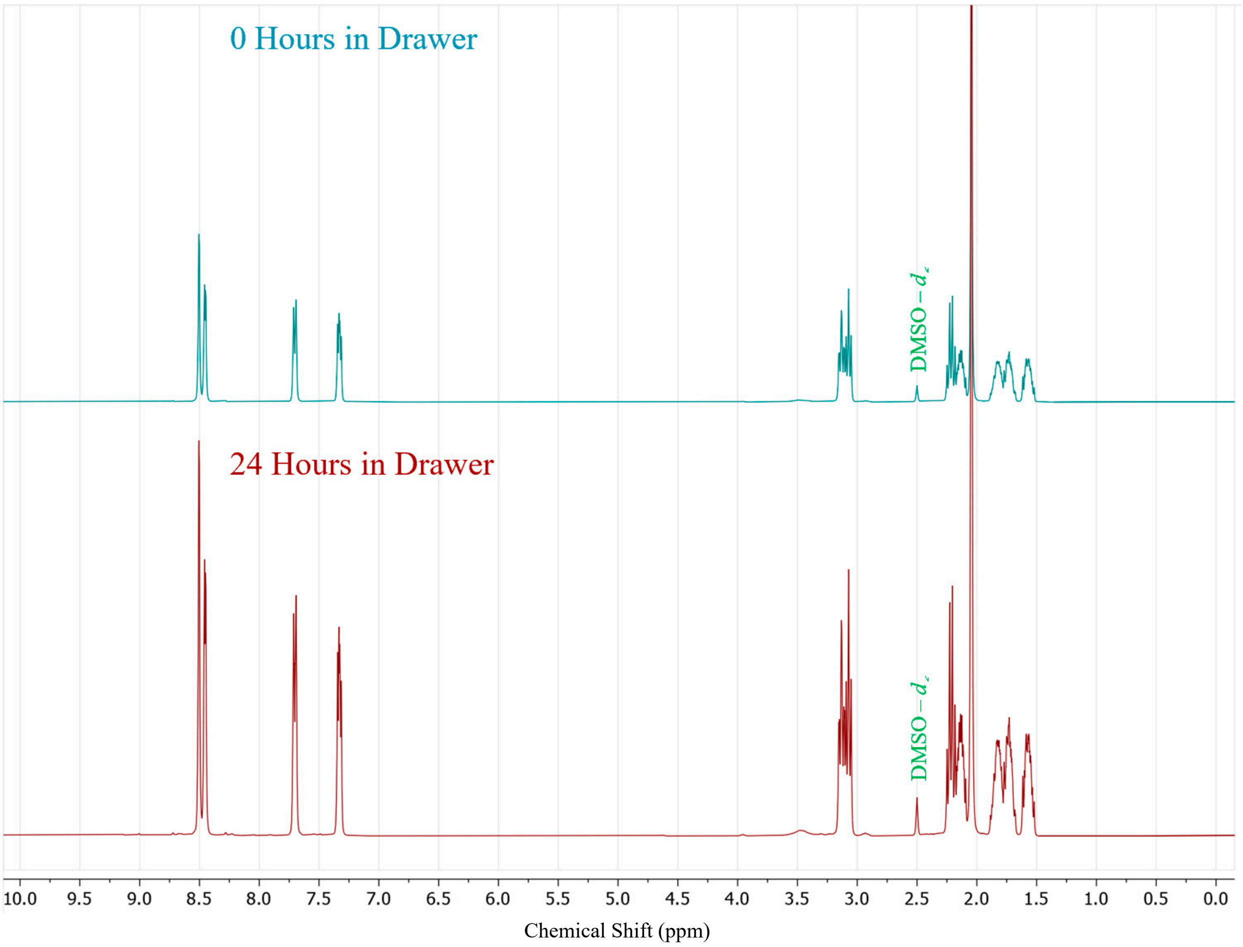

Figure S7: ^{1}H-NMR spectra of solutions of (S)-nicotine prepared from samples in which the liquid was in a vial in a dark drawer for either 0 hours (upper) or 24 hours (lower).

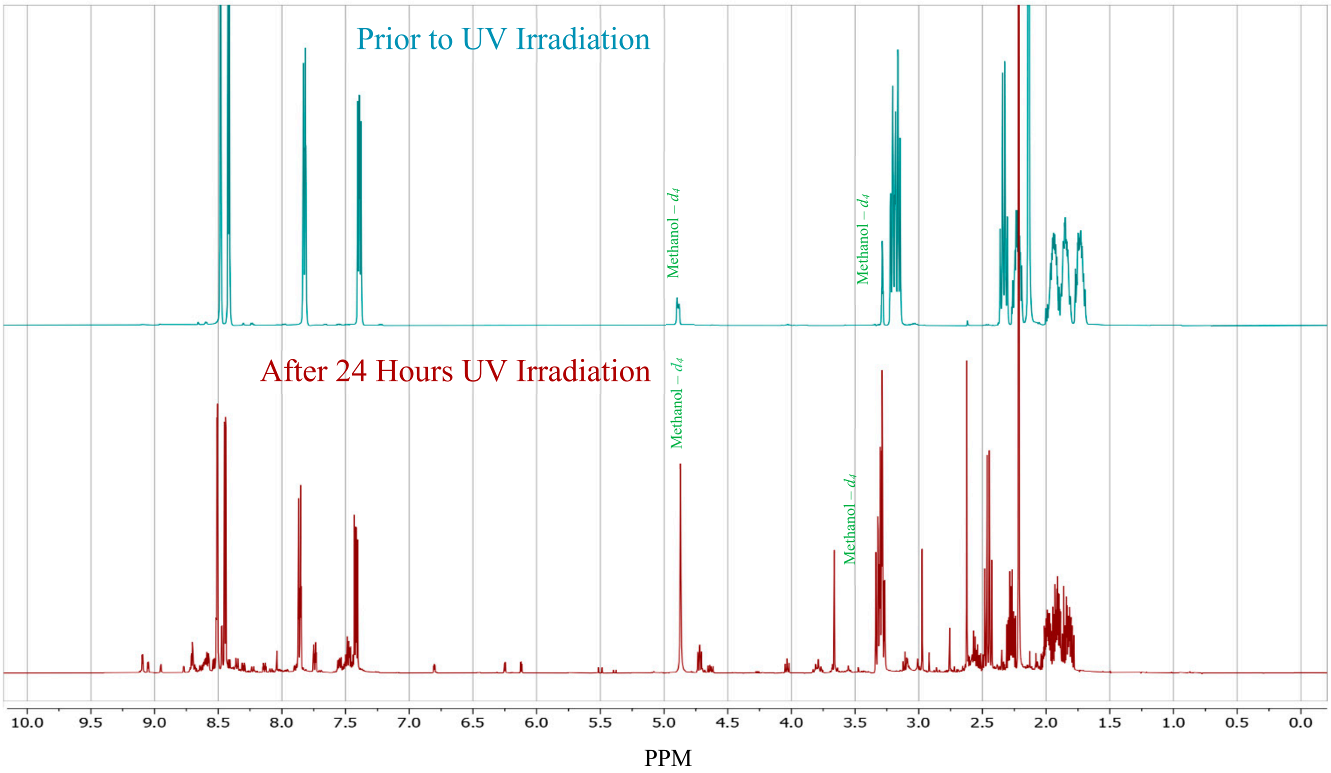

Figure S8: ^{1}H-NMR spectra of deuterated methanol solutions of liquid (S)-nicotine prepared from samples in which the liquid API received either no UV irradiation (upper) or 24 hours of UV irradiation (lower).

Hirshfeld Interaction Percentages

Table S3: The Hirshfeld surface computed interaction percentages for the API in the gentisate salt and in the other previously reported nicotinium salts.

Interaction Type	Gentisate	Orotate	DL-Malate	D-Malate	L-Malate II	L-Malate I
**O-H/H-O**	16.7	25.8	24.7	25.4	24.8	24.5
**H-H**	49.9	52.8	51.6	51.8	50.8	52.9
**H-C/C-H**	20.5	7.4	10.3	12.7	12.3	9.4
**H-N/N-H**	10.2	6.4	10.6	10.1	10.7	10.5
**O-C/C-O**	0.8	1.2	1.0	0.0	1.0	1.0
**C-C**	1.3	2.8	0.0	0.0	0.0	0.1
**C-N/N-C**	0.0	3.0	1.8	0.0	0.0	1.6
**N-O/O-N**	0.6	0.2	0.0	0.0	0.4	0.0
**N-N**	0.0	0.4	0.0	0.0	0.0	0.0

Thermal Properties

Table S4: Thermodynamic properties of (S)-nicotinium gentisate.

Stuart SMP10 Melting Point (°C)	DSC Endotherm Melting Point (°C)	$\Delta H^{\circ}_{fusion}$ (J/g)	$\Delta H^{\circ}_{fusion}$ (kJ mol^{-1})	$\Delta S^{\circ}_{fusion}$ (kJ mol^{-1} K^{-1})
151-155	155.9	94.74	29.97	6.986×10^{-2}

IR Spectra

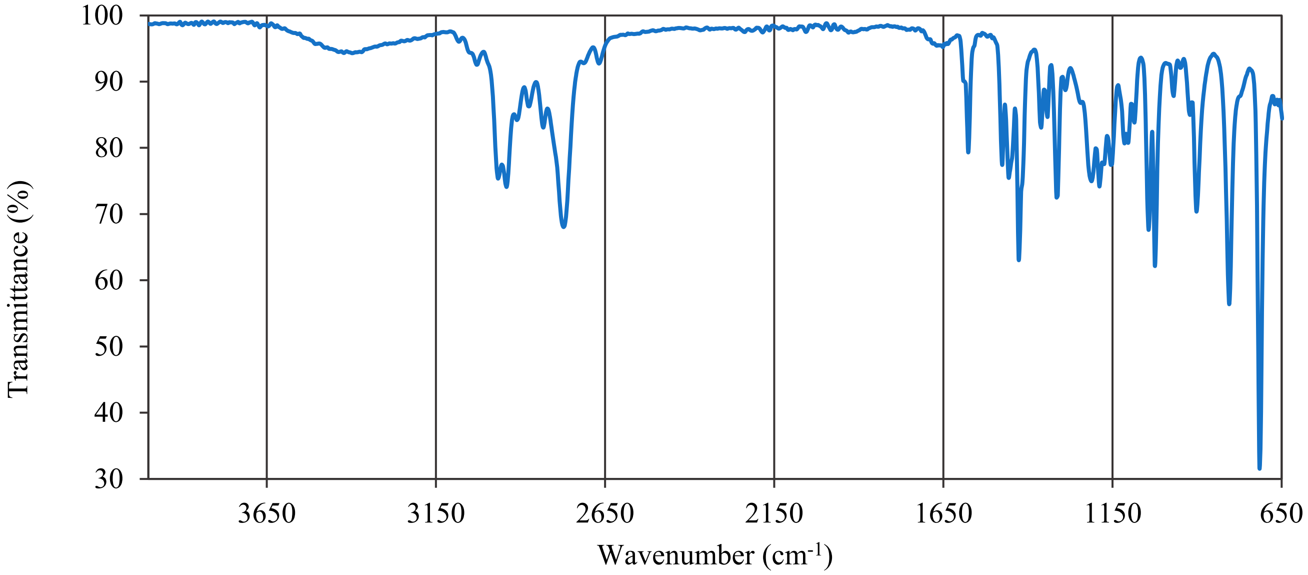

Figure S9: Infrared (IR) spectrum of (S)-nicotine.

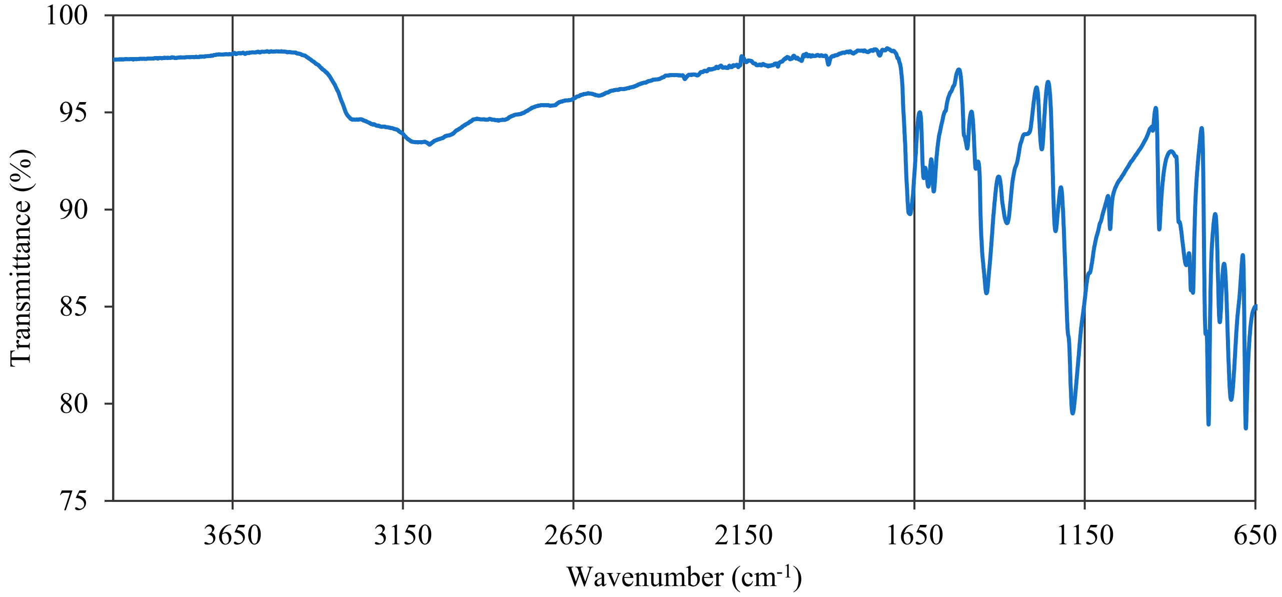

Figure S10: Infrared (IR) spectrum of gentisic acid.

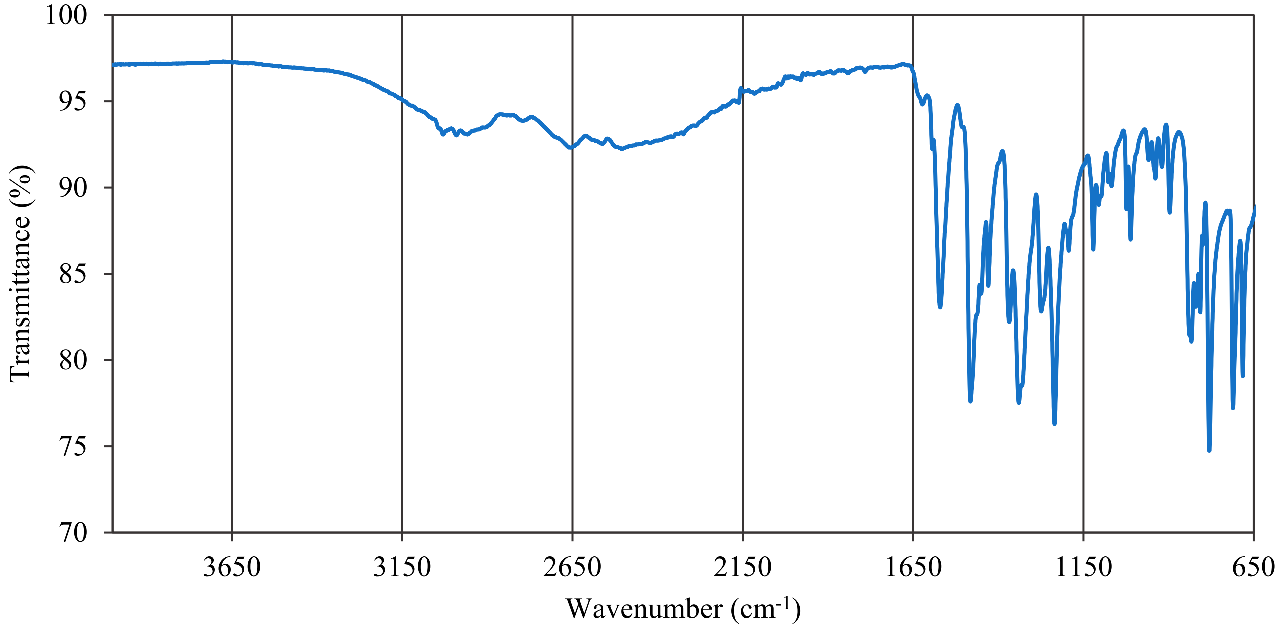

Figure S11: Infrared (IR) spectrum of gentisate salt.

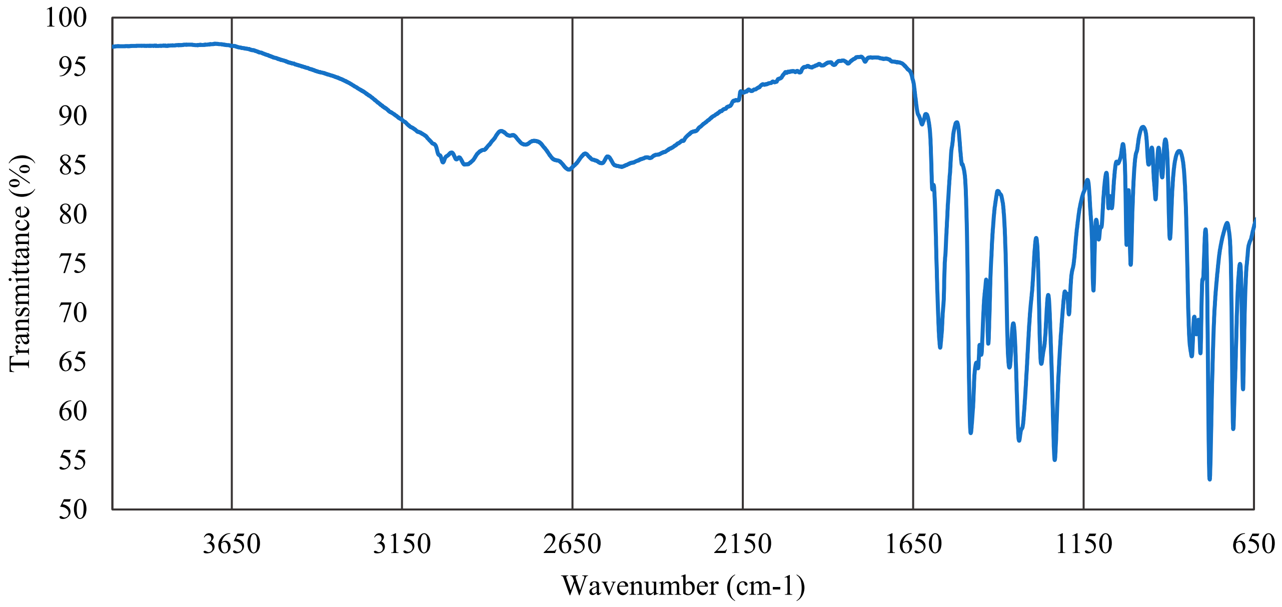

Figure S12: Infrared (IR) spectrum of gentisate salt melt.

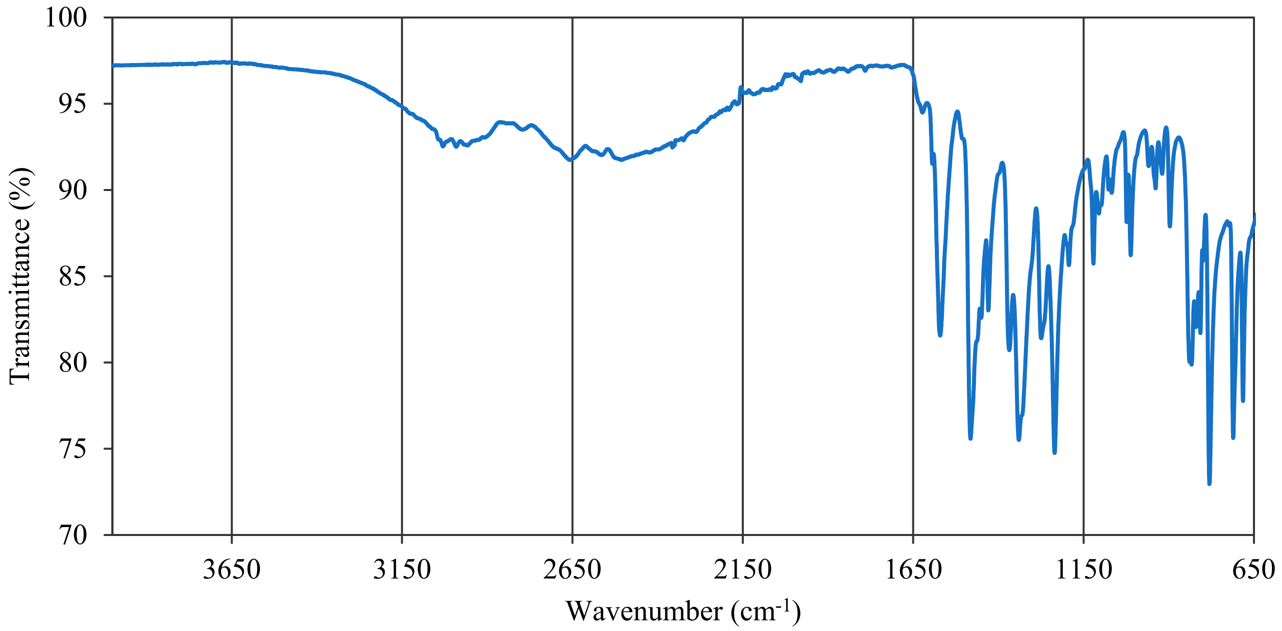

Figure S13: Infrared (IR) spectrum of gentisate salt after 24 hours of UV irradiation.

PXRD Before and After UV Irradiation

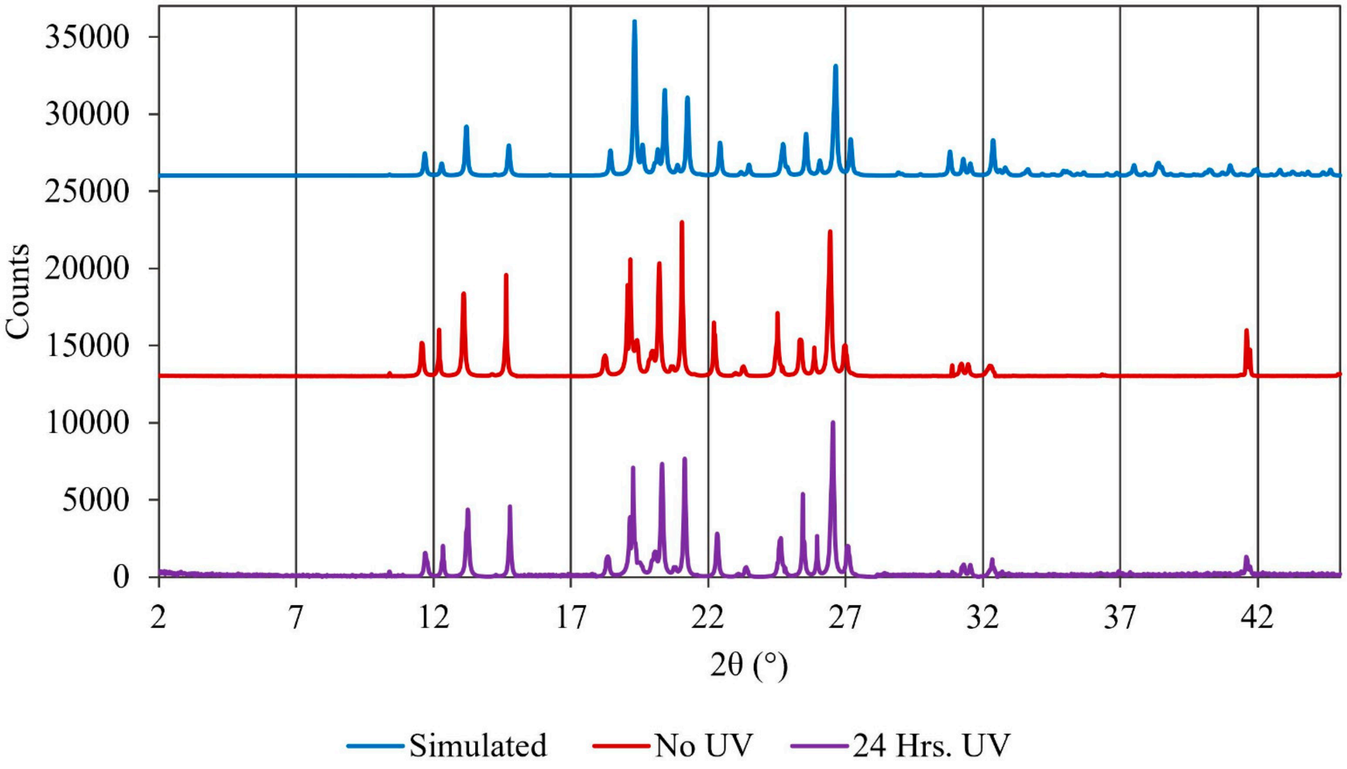

Figure S14: PXRD patterns of (S)-nicotinium gentisate simulated from SC-XRD analysis (upper), experimentally obtained prior to UV irradiation (middle) and after 24 hours of UV irradiation (lower). The pattern aquired prior to irradiation is offset by 13,000 counts and the Simulated pattern is offset by 26,00 counts.
